# Changes in Lipid Profile Secondary to Asymptomatic Malaria in Migrants from Sub-Saharan Africa: A Retrospective Analysis of a 2010–2022 Cohort

**DOI:** 10.3390/tropicalmed10050134

**Published:** 2025-05-15

**Authors:** Diego Gayoso-Cantero, María Dolores Corbacho-Loarte, Clara Crespillo-Andújar, Sandra Chamorro-Tojeiro, Francesca Norman, Jose A. Perez-Molina, Marta González-Sanz, Oihane Martín, José Miguel Rubio, Beatriz Gullón-Peña, Laura del Campo Albendea, Rogelio López-Vélez, Begoña Monge-Maillo

**Affiliations:** 1 National Referral Unit for Tropical Diseases, Infectious Diseases Department, Ramón y Cajal University Hospital, Instituto Ramón y Cajal de Investigación Sanitaria (IRYCIS), 28034 Madrid, Spain; diego.gayoso@salud.madrid.org (D.G.-C.); clara.crespillo@salud.madrid.org (C.C.-A.); sandra.chamorro@salud.madrid.org (S.C.-T.); francesca.norman@salud.madrid.org (F.N.); jperezm@salud.madrid.org (J.A.P.-M.); marta.gonzs@salud.madrid.org (M.G.-S.); beatriz.gullon@saludentreculturas.es (B.G.-P.); rogelio.lopezvelez@salud.madrid.org (R.L.-V.); 2 Health Sciences, University of Alcalá, 28871 Alcalá de Henares, Spain; 3 CIBER de Enfermedades Infecciosas, Instituto de Salud Carlos III, 28029 Madrid, Spain; oihane.martin@salud.madrid.org; 4 Microbiology Department, Ramón y Cajal University Hospital, IRYCIS, 28034 Madrid, Spain; 5 National Centre for Microbiology, Instituto de Salud Carlos III, 28029 Madrid, Spain; jmrubio@isciii.es; 6 Clinical Biostatistics Unit, Ramón y Cajal University Hospital, IRYCIS, 28034 Madrid, Spain; lcampo@salud.madrid.org; 7 CIBER Epidemiología y Salud Pública (CIBERESP), 28029 Madrid, Spain

**Keywords:** malaria, *Plasmodium*, asymptomatic infection, lipid profile, cholesterol, Sub-Saharan Africa

## Abstract

Altered lipid profiles have been observed in acute malaria, though mechanisms remain unclear. The impact of asymptomatic submicroscopic malaria infection (AMI) on lipids is unexploredAn observational, comparative, retrospective study was conducted of 1278 asymptomatic Sub-Saharan African migrants (ASSAMs) screened for malaria and lipid profiles during health exams (2010–2022). A systematic screening protocol for infectious disease was performed, including screening for *Plasmodium* spp. infection by polymerase chain reaction (PCR).Among 800 ASSAMs screened for malaria, 104 (13%) were PCR-positive: *P. falciparum* (68.72%), *P. malariae* (18.27%), *P. ovale* (9.62%), and mixed infections (3.8%). Participants with AMIs exhibited lower baseline lipid levels: total cholesterol (146 vs. 163 mg/dL; *p* < 0.001), HDL (43 vs. 47 mg/dL; *p* < 0.001), and LDL (87.5 vs. 98 mg/dL; *p* < 0.001), with no differences in triglycerides. After treatment, lipid levels partially equalized: total cholesterol (156 vs. 166; *p* = 0.01), HDL (44 vs. 47.5; *p* = 0.05), LDL (102 vs. 108.5; *p* = 0.31), with no changes in triglycerides. Patients with AMI showed higher rates of co-infections (Strongyloides 20.61% vs. 14.35%; *p* < 0.001; filariae 7.69% vs. 1.91%; *p* = 0.02) and lower mean corpuscular volume (87.2 vs. 85; *p* < 0.001). Conclusions: These findings suggest that cholesterol reductions in AMI are not solely due to acute inflammation but may reflect chronic inflammatory processes triggered by asymptomatic malaria. This supports a potential link between AMI and lipid profile changes, underscoring its role in subclinical chronic inflammation.

## 1. Introduction

Malaria is an infectious disease caused by parasites of the Plasmodium genus, members of the phylum Apicomplexa. It is the most significant parasitic disease worldwide in terms of morbidity and mortality. Although malaria incidence has declined over the past decade, approximately 247 million people across 84 endemic countries were diagnosed in 2022. Globally, in 2023, the number of malaria cases was estimated at 263 million, with an incidence of 60.4 cases per 1000 population at risk. This is an increase of 11 million cases from the previous year and a rise in incidence from 58.6 cases per 1000 population at risk in 2022 [1]. While Europe is non-endemic for malaria, 6131 cases were reported in the EU/EEE in 2022, of which 5375 cases had a known importation status [2].

The clinical spectrum of malaria ranges from asymptomatic cases, common among semi-immune migrant patients, to severe infections that can lead to septic shock and death [3]. Changes in lipid profiles have been observed in severe malaria and sepsis, conditions often characterized by systemic inflammation. In sepsis, organ failure is associated with reduced plasma cholesterol levels, including total cholesterol, LDL, HDL, and elevated triglycerides (TGs) [4]. Whether these abnormalities are caused by an increased clearance of lipoproteins or reduced synthesis and secretion of their precursors in the liver during acute disease remains elusive [5]. This reflects the interaction between inflammation and cholesterol metabolism, possibly driven by immune system activity and neuroendocrine-mediated metabolic changes to optimize energy use during disease [6].

Similar lipid abnormalities are seen in acute malaria, irrespective of the *Plasmodium* species, patient age, or origin. Reduced levels of cholesterol (total, LDL, and HDL) and elevated TGs are common findings [7,8]. Some researchers attribute these changes to acute inflammation, such as that occurring in sepsis [9]. The subendothelial retention of HDL cholesterol and altered bile salt and cholesterol production may underlie these changes. However, the lipid alterations in malaria appear more pronounced than in other infections, suggesting a specific interaction between *Plasmodium* and cholesterol [10,11]. Indeed, various studies conducted in endemic regions of malaria indicate that hypocholesterolemia may exert protective effects against *Plasmodium* infection [12].

This association may be explained by the fact that, although the phylum Apicomplexa is completely cholesterol-dependent, it lost its ability to produce cholesterol early during evolution. As a result, *Plasmodium* relies on cholesterol from its host [13]. This partially explains that *Plasmodium* spp. specifically invade hepatocytes and erythrocytes to proliferate. Hepatocytes are one of the most specialized cells in the internalization of glucose and cholesterol, by which free cholesterol is released and available for the parasite. In turn, erythrocytes are characterized by containing more than 50% of circulating cholesterol.

In vitro studies show that lowering serum cholesterol reduces *Plasmodium*’s ability to invade hepatocytes [13]. Additionally, the parasite’s invasion of erythrocytes reduces cholesterol in erythrocyte membranes, which may help evade immune responses by making erythrocytes less susceptible to monocyte and hemolysin attacks [14,15].

Clinically, hypercholesterolemia has been linked to severe malaria, potentially due to comorbidities like microangiopathy [16]. However, other authors suggest that high cholesterol levels could increase cholesterol availability for the parasite, thereby raising parasitemia. The exact cholesterol threshold above which malaria becomes severe remains unclear [17]. Lower cholesterol levels have been documented for asymptomatic individuals with submicroscopic parasitemia, but evidence in this area is limited [18].

During the process of parasite entry into the infected cell, an invagination of the cell membrane components occurs to create the parasitophorous vacuole membrane. Among the components that are carried along is membrane cholesterol, and this change in cholesterol composition may influence the biophysical and/or mechanical properties of the cell membrane [15]

During its intraerythrocytic growth, *P. falciparum* digests hemoglobin, exposing itself to a highly oxidative environment rich in iron and heme, leading to oxidative stress. This stress causes lipid peroxidation, primarily affecting unsaturated membrane phospholipids and posing toxicity to cells. Host antioxidant enzymes play a key role in repairing this damage by replacing oxidized lipids and restoring membranes, such as peroxiredoxin 6 (PRDX6) [19].

Oxidative stress is a key factor in malaria, and 4-Hydroxynonenal (4HNE) is a major byproduct of this process. This molecule can diffuse in and out of cells and has been shown to weaken the immune function of phagocytic cells, disrupt the maturation of red blood cells, and accelerate their removal by the spleen, contributing to anemia. During malaria, 4HNE levels are elevated as a result of the lipid peroxidation [20].

To the best of our knowledge this study presents the largest series ever published involving asymptomatic malaria patients outside an endemic area over 12 years, in which the lipid profile was determined in relation to infection, and analyzing the potential lipid changes during treatment and the influence of associated coinfections.

## 2. Materials and Methods

### 2.1. Study Design and Participants

An observational, retrospective analysis was conducted at the National Referral Unit for Tropical Medicine (NRU-Trop) of Ramón y Cajal University Hospital in Madrid, Spain, between January 2010 and December 2022.

#### 2.1.1. Study Population

The patients included in the study were asymptomatic Sub-Saharan migrants (ASSAMs) older than 16 years admitted to the NRU-Trop for a health exam. Patients visiting the NRU-Trop are referred by Primary Care, medical specialists, or non-governmental organizations (NGOs). Other patients visit the NRU-Trop of their own initiative, motivated by their symptoms or need for a health screening.

Asymptomatic patients were categorized into two groups according to their malaria test results, as follows:

- Patients with asymptomatic malaria infection (AMI): without any symptoms suggestive of malaria (fever, cephalea, myalgias, arthralgias, or other symptoms that patients associate with previous malaria infections) or any previous treatment for malaria within the past three months and with a positive polymerase chain reaction (PCR) result for *Plasmodium* spp.
- Patients without asymptomatic malaria infection (non-AMI): without any symptoms suggestive of malaria and with a negative PCR for *Plasmodium* spp.

#### 2.1.2. Variables Collected

Demographic and epidemiological variables: date of birth; age; sex; country of birth; pre-consultation period (defined as the number of months from arrival in Spain to first consultation with NRU-Trop); and duration of the migration journey (defined as the number of months from departure from the country of origin to arrival in Spain).

Analytical variables: serum biochemistry; lipid profile (total cholesterol, LDL, HDL and TGd, using the Alinity C equipment from Abbot Diagnostics for the lipid profile determinations); complete blood count; and hemoglobinopathies if clinically suspected. Microbiological variables: determinations were performed according to the NRU-Trop protocol for asymptomatic Sub-Saharan migrant patients [21], including HIV, HBV, and HCV serology; syphilis, (all carried out by Architect^®^ Abbott, Abbott Park, Illinois); *Strongyloides* spp. and *Schistosoma* spp. (both performed on NovaLisa^®^ Novatec Immundiagnostica GmbH); and stool ova and parasite exam. Patients with suspicious symptoms or eosinophilia were also evaluated for blood and skin microfilariae. Screening for malaria was performed using PCR to detect *Plasmodium* spp. DNA in peripheral blood. Genomic DNA was extracted from 200 μL of whole blood collected in EDTA tubes using the QIAamp DNA Mini Kit (QIAGEN^®^, Hilden, Germany), following the manufacturer’s protocol.

The detection and species identification of *Plasmodium* spp. were carried out using a nested multiplex PCR [22]. This approach consists of two sequential multiplex PCRs targeting the small subunit ribosomal RNA (ssrRNA) gene of Plasmodium. The first round amplifies a conserved region of Plasmodium spp. along with an internal control to ensure reaction validity. The second round enables species-level differentiation based on the distinct fragment sizes observed after agarose gel electrophoresis.

During the study period, two different PCR techniques were used. Until December 2017, in-house multiplex nested PCR was used, which identifies the four species of malaria in humans (*P. falciparum, P. vivax, P. ovale*, and *P. malariae*) in two serial amplification assays. From January 2018, screening was carried out using a commercially available multiplex real-time PCR (*Plasmodium* Typing Real-Time PCR, Bio-Evolution^®^, Bussy-Saint-Martin, France) [23].

Monitoring variables: in patients with a positive PCR for malaria, laboratory tests were repeated 4 or 6 weeks after treatment completion to confirm the negative PCR result. Patients negative for malaria or positive for malaria but with other concomitant conditions underwent the appropriate control laboratory tests. Biochemistry and lipid profiles were generally included. Other variables collected included time from consultation to first laboratory testing and time between the first and second laboratory test (in weeks).

An analysis of sensitivity of cholesterol values was subsequently performed based on coinfections by all those with statistically significant differences between the two groups.

#### 2.1.3. Statistical Analysis

Descriptive statistics were used to summarize the epidemiological and analytical characteristics of patients. Continuous variables were expressed as medians and interquartile ranges (IQRs) and as means and standard deviation (SD). Categorical variables were expressed as absolute frequencies and percentages. Student’s *t*-test and the Mann–Whitney U test were used to assess differences between continuous variables according to their distribution. The association between categorical variables was assessed by the chi-squared test or Fisher’s exact test, as appropriate. A *p* value < 0.05 was considered statistically significant. Statistical analyses were performed using STATA ^®^ version 16.1 (StataCorp LP, College Station, TX, USA).

An analysis of sensitivity of cholesterol values based on coinfection by *Strongyloides* spp. was subsequently performed.

Finally, the Wilcoxon signed-rank test was performed to estimate median values for paired samples and compare pre- and post-treatment lipid profile values in the AMI group.

### 2.2. Ethical Aspects

This study was approved by the Ethics Committee of Ramon y Cajal Hospital (179/14). Initial approval: 15 June 2014. Protocol update: 15 May 2021.

## 3. Results

**Figure 1:**
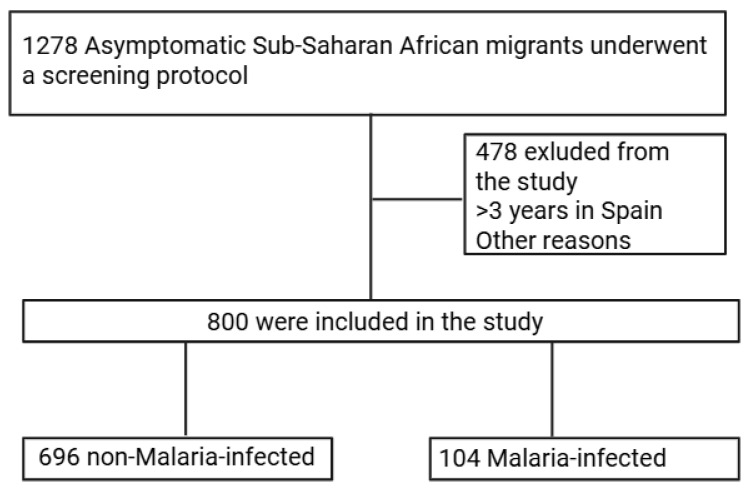
Flow chart Malaria screening.

During the study period, a total of 1278 ASSAM patients were examined; among these, 800 (62.6%) patients who had a PCR test for malaria were included. In total, 104 PCR tests were positive for malaria, yielding a prevalence of AMI of 13% (Figure 1).

Of the 800 patients included in the study, 690 (86.25%) were male. Notably, the patients with a positive PCR for malaria were younger than those with a negative PCR for malaria, with a mean age of 24.5 years vs. 27.1 years (*p* = 0.017), respectively. Differences were also observed in relation to the median transit time from the country of origin to Spain, which was lower in the AMI group, 3 vs. 4 months (*p* = 0.003), respectively. The median pre-consultation time was also lower in the AMI group, 3 vs. 4.5 months (*p* = 0.003), respectively. No differences were found between the groups regarding the time from consultation to first laboratory testing (Table 1).

In connection with the origin of patients, 523/800 (65.4%) were from Western Africa, 254/800 (31.8%) from Central Africa, 16/800 (2%) from Eastern Africa, and 7/800 (0.8%) from Southern Africa (Supplementary Table S1).

### 3.1. Results of Screening for Malaria

The *Plasmodium* species identified were *P. falciparum* in 70/104 (67.3%) cases; *P. malariae* in 19/104 (18.3%); *P. ovale* in 10/104 (9.6%); and mixed infections in 5/104 (4.8%) cases. Most cases of malaria were diagnosed within the first 12 months from arrival in Spain (91/104, 87.5%) (Table 2). The anti-malaria treatment most frequently used was Autovacuona-proguanil (54/104, 52%). A total of 16.3% of patients were lost to follow-up and did not receive any treatment (Supplementary Table S2).

### 3.2. Results of Screening for Other Infectious Diseases

Only those results of the screening in which significant relationships were found with respect to the lipid profile were included. HBV, *Strongyloides* spp., and filariae infection rates had statistically significant differences between the two groups (Table 3). The prevalence of previous HBV infection was significantly higher in patients with AMI (50% vs. 37.4%; *p* < 0.001). The prevalence of infection by *Strongyloides* spp. was also higher in AMI patients (41.3% vs. 20.3%; *p* < 0.001). The determination of filariae was not performed in all participants, only in those with clinical suspicion. The incidence of filariae was higher in AMI patients (8/35, 22.9%) compared to non-AMI patients (13/181, 7.2%) (*p* = 0.004).

### 3.3. Hemogram and Screening for Hemoglobinopathies

This test was requested only for those with clinical suspicion. There was no difference between the two groups related to the prevalence of hemoglobinopathies such as thalassemias and abnormal HbA. The prevalence was 33.3% in AMI patients (9/27) compared to 21.4% in those without malaria (6/28) (*p* = 0.32; SMD 0.264). Only one AMI patient had G6PD deficit. Erythrocyte size (fL) was higher in non-AMI (87.1 vs. 84.4; *p* < 0.001).

### 3.4. Lipid Profile

The lipid profile from the first visit was available for 772 of the 800 patients who underwent screening for malaria (676 non-AMI and 96 AMI). The median total cholesterol (TC) was significantly lower in AMI patients (146 mg/dL) vs. non-AMI patients (163 mg/dL) (*p* < 0.001). HDL and LDL were tested in 543 non-AMI and 74 AMI patients. Median HDL and LDL values were significantly lower in AMI patients (HDL 43 mg/dL vs. 47 mg/dL; LDL 87.5 mg/dL vs. 98 mg/dL; *p* < 0.001). There were no statistically significant differences in triglycerides between the two groups: 65 mg/dL for AMI patients and 64 mg/dL for non-AMI patients (*p* = 0.744).

A second laboratory analysis, including lipid profile, was performed during follow-up at the treating physician’s discretion. In the case of AMI patients, analysis was performed to confirm that malaria PCR was negative six weeks after treatment, but not all physicians included a lipid profile. In the case of non-AMI patients, the second laboratory analysis was carried out for other reasons, and lipid profile was not always included. No significant differences were observed in the period between laboratory tests: a median of 10.9 weeks in AMI patients [6.9; 17.1] vs. 11 weeks in non-AMI [7.1; 14.0] (*p* = 0.523).

During follow-up, TC was included in the second laboratory analysis in 260 non-AMI and 49 AMI patients. The median TC was significantly lower in AMI-treated patients as compared to the non-AMI group: 156 mg/dL vs. 166 mg/dL (*p* = 0.003). Regarding TGs, no significant difference was observed.

HDL and LDL cholesterol was included in 133 non-AMI patients and 24 AMI patients. Significant differences between medians disappeared for LDL: HDL 44 mg/dL for AMI treated patients vs. 47.5 mg/dL for non- AMI (*p* = 0.048); LDL 102 mg/dL for patients with treated AMI vs. 108.5 mg/dL without previous AMI (*p* = 0.208). Differences remained non-significant in triglyceride levels, with a median of 63 mg/dL for the two groups (*p* = 0.811) (Table 4). Regarding HDL values, although statistical significance is still present, it lies at the threshold of significance, having initially started with a *p*-value < 0.001. Therefore, based on the observed trend, it is likely that this statistical significance would eventually disappear over time.

TC was included in the first and second laboratory tests in 47 AMI patients. The median TC was 144 mg/dL in the first test and 156 mg/dL in the second test. The Wilcoxon test for paired medians revealed a statistically significant increase between the first and the second determination (*p* = 0.038). In the non-AMI group, TC was requested in 249 patients in the first and second determination, with a median of 191 and 192 mg/dL, respectively (*p* = 0.710).

LDL-cholesterol was included in the first and second tests for 20 AMI patients, with a median of 89 mg/dL in the first test and 105 mg/dL in the second. Although this difference is not statistically significant (*p* = 0.135), it is clinically relevant, with a difference of 16 mg/dL between the two tests. LDL cholesterol was measured in the first and second tests in 106 non-AMI patients, increasing only from 103 mg/dL to 109 mg/dL (*p* = 0.217.)

In relation to HLDc in the AMI group, it was tested twice in 20 patients, with a mean of 39 mg/dL at baseline and 44 mg/dL after treatment completion. This difference shows a borderline significance (*p* = 0.059). In the non-AMI group, HDLc was tested twice in 106 patients, with a mean of 50 mg/dL at baseline and 48.5 mg/dL on treatment completion (*p* = 0.310).

**Figure 2:**
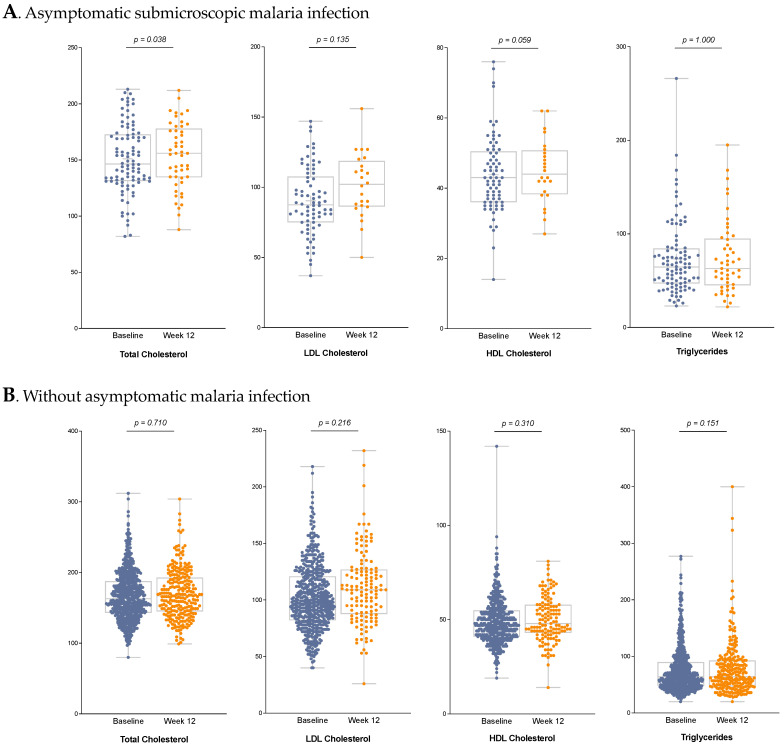
Comparisons of total cholesterol, LDL cholesterol, HDL cholesterol, and triglycerides in the asymptomatic submicroscopic malaria infection (AMI) group (**A**) and the non-AMI group (**B**) at baseline (blue) and at 12 weeks (orange). Each point represents an individual data value. The box in each box-and-whisker plot displays the interquartile range, with the horizontal line indicating the median; whiskers extend to the minimum and maximum values within 1.5 times the interquartile range. Statistical comparisons were performed using the Wilcoxon signed-rank test for paired measurements; *p*-values are shown above each pair of boxplots, and *p* < 0.05 was considered statistically significant.

No statistically significant differences were observed in levels of TGs among the 47 AMI patients who were tested before and after treatment (68 mg/dL vs. 63 mg/dL; *p* = 0.999) or among the 250 non-AMI patients who had the two tests (61 mg/dL vs. 63.5 mg/dL; *p* = 0.151) (Figure 2).

## 4. Discussion

We found significant differences in TC, HDL, and LDL levels in patients with asymptomatic malaria without changes in TG levels. These findings suggest a relationship between asymptomatic malaria and lipid abnormalities. Changes in lipid profiles after anti-malarial treatment point to a cause–effect relationship between the two.

Traditionally, lipid changes in malaria were attributed to acute inflammation. However, distinctive aspects of malaria, including asymptomatic cases, may explain this association. The lack of TG changes, commonly seen in inflammation, suggests a different origin for the cholesterol abnormalities than acute inflammation, potentially linked to the parasite’s cholesterol consumption for survival. The cholesterol composition in the membrane of infected erythrocytes affects their ability to escape the immune system. The primary source of cholesterol in the infected erythrocyte membrane is the growth medium plasma (lipoproteins) [24,25]. The influence of plasma cholesterol on the erythrocyte membrane may explain the correlation between nutritional status and the severity of malaria [26] and the fact that metabolic changes due to nutrition could influence resistance to treatment in animal models [24].

Patients with negative PCR results had lived longer in Spain than those with positive results, which could be due to a longer period for clearing a possible submicroscopic infection. This difference in the period since arrival could also affect the migrants’ dietary habits and may influence their lipid profiles. However, the difference was only of 1.5 months, and 90% of the patients undergoing screening had been in Spain for less than 12 months. This likely limits substantial dietary impact. Furthermore, we should not assume that the dietary habits of patients were more heart-healthy in their country of origin. In fact, cardiovascular diseases are prevalent in malaria-endemic regions, and 75% of global mortality from such reasons occurs in these endemic areas [27,28]. Although regular use of this medication was not collected in our study, differences in cholesterol levels are unlikely due to lipid-lowering therapies, as most patients were young (average age < 28 years) and unlikely to have been tested or treated for cholesterol in their home countries.

Co-infections with *Strongyloides* spp., filariae, or previous HBV infection were more common in AMI cases. This could be due to the higher prevalence of these conditions in the countries of origin of AMI patients, where poor sanitary conditions and limited mass drug administration for parasitic infections may converge. Regarding specifically *Strongyloides*, its chronic infection may influence the inflammatory response against *Plasmodium* and affect parasitic load [29,30,31]. Moreover, *Strongyloides* spp. infection has been associated with lower cholesterol levels, likely due to malnutrition, altered intestinal lipid production, and genetic factors [32]. However, in our study, AMI and non-AMI patients exhibited different metabolic profiles, irrespective of the presence or absence of *Strongyloides* spp. infection and the treatment administered.

Abnormal erythrocyte size in malaria is attributed to changes in erythrocyte membrane rigidity, adhesiveness, and permeability induced by the parasite, which may explain the abnormal size of erythrocytes in our series [33,34]. Hemoglobinopathies, such as HbAS, α-thalassemia, and sickle cell anemia, provide resistance to malaria, explaining their prevalence in endemic areas [35,36,37]. A correlation has been observed between the presence of hemoglobinopathies and lower cholesterol levels, which may be explained by the presence of associated mutations in genes related to lipid metabolism, as well as by environmental factors [38]. Chronic anemia due to hemoglobinopathies increases because of a higher erythropoietic activity, which could explain the lipid differences observed in other studies [39]. Therefore, it seems that in the presence of hemoglobinopathies, lower cholesterol levels may appear. However, in our study, we did not find a higher presence of hemoglobinopathies in patients with malaria that could act as confounders in the cholesterol differences.

Lipid profiles improved in asymptomatic malaria patients following anti-malarial treatment, but total cholesterol levels remained lower than in uninfected patients. Of note, other lipid parameters increased to equal the levels of non-AMI patients. This finding could be related to the fact that, apart from HDL and LDL cholesterol, total cholesterol includes other fractions that could be potentially abnormal but were not measured in our study and are not routinely measured in clinical practice. This finding gives rise to the theory that AMI patients may have underlying hypocholesterolemia, suggesting a protective effect against severe symptomatic malaria [40]. Genetic polymorphisms linked to hypocholesterolemia correlate with mild malaria and reduced mortality [3,41], whereas other polymorphisms related to hypercholesterolemia are associated with severe disease and higher parasitemia [42].

The lipid profile variations in asymptomatic malaria reflect the dynamic host–parasite relationship. Recurrent malaria depletes lipid reserves needed for parasite membrane development and also affects the cholesterol reserve for the immune system leading to equilibrium between parasite and host during AMI, suggesting an adaptive competence for survival [43]. On another note, cholesterol may play a role in immunotolerance to malaria in asymptomatic patients.

It is debated whether submicroscopic malaria should be classified as asymptomatic, as it has been observed to have significant implications for patients who experience it. A study conducted in an endemic area showed that asymptomatic patients with malaria consistently exhibit elevated levels of pro-inflammatory cytokines, in continuous balance with anti-inflammatory or regulatory cytokines [44].

There is an established relationship between inflammation and cholesterol metabolism. Hypercholesterolemia leads to cholesterol accumulation in macrophages and other immune cells, which, in turn, promotes inflammatory responses. Cholesterol metabolism plays a key role in the immune response and in the organism’s ability to clear infections [45].

Disruptions in cellular or organismal cholesterol homeostasis, which occur as part of innate immune responses, can enhance inflammatory responses through mechanisms like TLR signaling or inflammasome activation. This physiological adaptation, exemplified by HDL-mediated cholesterol efflux and reverse cholesterol transport (RCT), becomes dysfunctional in chronic metabolic diseases. This dysfunction may explain why chronic infections and autoimmune disorders are often associated with reduced levels of HDL, increased levels of atherogenic lipoproteins, and accelerated atherosclerosis [46]. Submicroscopic malaria, as a chronic disease, could be affected in the same way, explaining the cholesterol changes observed.

Lipid peroxidation disrupts membrane organization, altering fluidity, permeability, metabolic processes, and ion transport, and can modify LDL, making it pro-atherogenic. In erythrocytes, lipid oxidation occurs more extensively than in plasma [47].

Cholesterol is less affected by lipid peroxidation than polyunsaturated fatty acids (PUFAs). The oxidizability of lipids decreases with fewer double bonds, meaning that cholesterol is oxidized only after most unsaturated fatty acids are depleted. It has been reported that during low-density lipoprotein (LDL) oxidation, cholesterol is oxidized only after most PUFA esters have been oxidized. Additionally, cellular membrane cholesterol is more susceptible to peroxidation than plasma cholesterol [48].

Although the peroxidation of plasma cholesterol could partly contribute to its reduction in the bloodstream, as oxidation products can be rapidly cleared from the blood, and lipid peroxidation could act as an inhibitor of cholesterol synthesis, their direct relationship has not been confirmed. Inflammation and oxidative stress may influence both processes, but further studies are needed to clarify their connection and potential causal mechanisms.

These possible inflammatory changes and the high prevalence of AMI observed in ASSAMs (13%) may highlight the need to consider screening migrants from endemic areas. Regarding when to perform the screening, although we set a three-year limit for malaria screening based on previous studies in our unit, most AMI cases were diagnosed within the first 12 months after arrival in Spain. This period would probably be more appropriate for screening. It is worth noting that one case was diagnosed 51 months after arrival, highlighting the possible long prevalence of Plasmodium in asymptomatic patients. In this case, possible autochthonous transmission was ruled out because there are no competent vectors in Spain capable of transmitting *Plasmodium* spp. (*Anopheles atroparvus*, *An. labranchiae*, and *An. sacharovi*). In addition, the patient had not received blood transfusions or organ transplants.

A retrospective design with a number of patients lost to follow-up are study limitations, as well as the change in molecular diagnostic technique during the study period, which could have introduced variability in the detection of *Plasmodium* species, despite both methods having comparable sensitivity and specificity. Another possible limitation of the study could be the considerable difference in sample sizes between study groups. However, appropriate statistical tests were selected based on the distribution and characteristics of the data—such as the use of non-parametric methods (e.g., Mann–Whitney *U* test and Wilcoxon signed-rank test) and exact tests (e.g., Fisher’s exact test)—which are robust to differences in sample size and reduce the potential for biased results. Therefore, the analytical strategy was specifically designed to account for and mitigate the impact of unequal group sizes, ensuring that the interpretation of results remains valid and statistically sound.

This is the first research to document lipid changes in asymptomatic malaria cases detected through routine screening outside endemic regions, minimizing bias from re-infections. This study contributes to understanding malaria pathophysiology, supporting the development of innovative approaches for diagnosis, treatment, and prevention.

## 5. Conclusions

This study demonstrates that lipid profile abnormalities are not only present in acute malaria but also in asymptomatic patients and that anti-malarial treatments also induce changes in lipid profile. Understanding the correlation between lipid profile and malaria infection is crucial. This highlights the importance of nutritional status in the progression of the infection, the influence of the lipid profile on the immune response, and the possible role of the lipid profile as a diagnostic and prognostic tool, as well as a potential treatment target using tools such as statins.

## Figures and Tables

**Table 1:** Demographic characteristics of asymptomatic patients with malaria and without asymptomatic malaria.

Variable	Non-Malaria Infection *N* = 696	Malaria Infection *N* = 104	*p*-Value
Gender, male %	596 (85.6)	94 (90.4)	0.333
Age, mean *	27.1 (10.4)	24.5 (8.8)	0.017
Pre-consultation period (months) Median **	4.5 [1.9; 9.5]	3.0 [1.1; 6.9]	0.003
Transient time (months) Median **	4 [1; 11]	3 [1; 8]	0.04
Time from consultation to laboratory testing (weeks) Median **	1.3 [0.9; 2.0]	1.1 [0.6; 2.1]	0.272
African endemic area (%) - West - Center - East - South	458 (65.8) 215 (30.9) 16 (2.3) 7 (1.0)	65 (62.5) 39 (37.5) 0 (0.0) 0 (0.0)	

* Standard deviation (SD) added in parentheses. ** The 25th percentile and 75th percentile [P25th; P75th] are added in square brackets.

**Table 2:** Number of cases and *Plasmodium* spp. species identified by pre-consultation time.

*Plasmodium* spp.	Pre-Consultation Time			Total
	<12 Months	12–24 Months	>24 Months	
*P. falciparum*	63	5	2	70 (67.3%)
*P. malariae*	15	1	3 *	19 (18.3%)
*P. ovale*	8	2	0	10 (9.6%)
*P. vivax*	0	0	0	-
*P. falciparum + P. ovale*	4	0	0	4 (3.8%)
*P. malariae + P. ovale*	1	0	0	1 (1%)
Total	91 (87.5%)	8 (7.7%)	5 (4.8%)	104 (100%)

* A case was diagnosed after a pre-consultation period of 51 months.

**Table 3:** Co-infections (only those with statistically significant differences between the two groups are included).

Variable	Non-Malaria Infection *N* = 696	Malaria Infection *N* = 104	*p*-Value
Hepatitis B Chronic infection Past infection Vaccinated Isolated anti-HBc Negative Not performed	109 (15.7) 260 (37.4) 24 (3.4) 92 (13.2) 182 (26.1) 29 (4.2)	14 (13.5) 52 (50.0) 3 (2.9) 10 (9.6) 23 (22.1) 2 (1.9)	0.252 *p* < 0.001 *
*Strongyloides* spp. Positive Negative Not performed	141 (20.3) 482 (69.3) 73 (10.5)	43 (41.3) 47 (45.2) 14 (13.5)	<0.001
Filariae Positive *Loa Loa* *Onchocerca* spp. *Mansonella* spp. Negative Not performed	13 (1.9) 5 (0.7) 1 (0.1) 7 (1.0) 168 (24.1) 515 (74.0)	8 (7.7) 2 (1.9) 0 (0.0) 6 (5.8) 27 (26.0) 69 (66.3)	0.004

* Regarding HBV, a statistically significant difference was found only for past hepatitis B infection (*p* < 0.001). For the six categories assessed collectively, no statistically significant differences were observed between the two groups (*p* = 0.252).

**Table 4:** Lipid profile results for asymptomatic patients with malaria and subjects without asymptomatic malaria at baseline and 12 weeks later.

Baseline Measurement				Control Measurement		
Variable Median	Non-Malaria Infection N = 676	Malaria Infection N = 96	*p*-Value	Non-Malaria Infection N = 260	Malaria Infection N = 49	*p*-Value
Total cholesterol * (mg/dL),	163 [143; 188]	146 [131; 173]	<0.001	166 [143.5; 192]	156 [135; 176]	0.003
HDL cholesterol (mg/dL), **	47 [41; 55]	43 [36; 50]	<0.001	47.5 [42; 57]	44 [38.5; 50.5]	0.048
LDL cholesterol (mg/dL), **	98 [83; 121]	87.5 [75; 107]	<0.001	108.5 [87; 127]	102 [86.5; 117.5]	0.208
Triglycerides (mg/dL), *	64 [47.5; 89]	65 [47; 85]	0.744	63 [47; 93]	63 [46; 94]	0.811

* CT and TG baseline measurement: 676 NMI patients and 96 AMI patients. During the measurement at 12 weeks: 260 NMI patients and 49 AMI patients. ** HDL and LDL baseline measurement: 543 NMI patients and 74 AMI patients. During the measurement at 12 weeks: 133 NMI patients and 24 AMI patients. The 25th percentile and 75th percentile [P25th; P75th] are added in parentheses.

## Data Availability

All data collected in the project was recorded and stored in an electronic data capture tool developed for this project under REDCap (Research Electronic Data Capture, Vanderbilt University, TN, USA). REDCap is a secure web application for managing online research databases. It facilitates collaborative work and is an optimal tool for clinical research, as its forms allow longitudinal follow-up. REDCap includes audit trails for tracking data manipulation and user activity, as well as tools for controlling access of users, and complies with national and international regulatory and legal requirements, including GDPR Regulation (EU) 2016/679. Due to the confidential nature of the data and the fact that participants only consented to their information being used within the project database, the datasets generated and/or analyzed during the current study are not publicly available. Data sharing is therefore restricted to protect patient privacy and comply with ethical approvals.

## Supplementary Materials

The following supporting information can be downloaded at: https://www.mdpi.com/article/10.3390/tropicalmed10050134/s1, Table S1: Country of origin of asymptomatic Sub-Saharan African migrants included in the study, Table S2: Characteristics of *Plasmodium* spp. infection–TREATMENT.
