# Supplementary material for: Changes in Lipid Profile Secondary to Asymptomatic Malaria in Migrants from Sub-Saharan Africa: A Retrospective Analysis of a 2010–2022 Cohort
Source: Trop Med Infect Dis. 2025 May 15;10(5):134. doi: 10.3390/tropicalmed10050134 (PMC12115369; doi:10.3390/tropicalmed10050134)
Supplement: Supplementary file 1 [file tropicalmed-10-00134-s001.zip › tropicalmed-3569235-supplementary.pdf]

SUPPLEMENTARY DOCUMENTS:

Suplementary Table S1. Country of origin of asymptomatic Sub-Saharan African migrants included in the study

**Country**	Malaria Infected	Malaria Non-Infected	Asymptomatic Sub-Saharan African migrants
-Western Africa	52 (62.5%)	458 (65.8%)	523 (65.4%)
Burkina Faso	7 (6.7%)	14 (2.0%)	21 (2.6%)
Cape Verde	0	1 (0.1%)	1 (0.1%)
Ivory Coast	19 (18.3%)	73 (10.5%)	92 (11.5%)
Gambia	0	18 (2.6%)	18 (2.3%)
Ghana	2 (1.9%)	8 (1.2%)	10 (1.3%)
Guinea-Bissau	2 (1.9%)	13 (1.9%)	15 (1.9%)
Guinea	23 (22.1%)	118 (17.0%)	141 (17.6%)
Liberia	0	1 (0.1%)	1 (0.1%)
Mali	8 (7.7%)	86 (12.4%)	94 (11.8%)
Mauritania	0	4 (0.6%)	4 (0.5%)
Niger	2 (1.9%)	2 (0.3%)	4 (0.5%)
Nigeria	1 (1.0%)	22 (3.2%)	23 (2.9%)
Senegal	0	89 (12.8 %)	89 (11.1%)
Sierra Leone	1 (1.0%)	9 (1.3%)	10 (1.3%)
-Central Africa	39 (37.5%)	215 (30.9%)	254 (31.8%)
Burundi	0	1 (0.1%)	1 (0.1%)
Cameroon	30 (28.9%)	155 (22.3%)	185 (23.1%)
CAR	1 (1.0%)	5 (0.7%)	6 (0.8%)
Chad	1 (1.0%)	6 (0.9%)	7 (0.9%)
Congo	1 (1.0%)	8 (1.2%)	9 (1.1%)
Equatorial Guinea	6 (5.8%)	36 (5.2%)	42 (5.3%)
Gabon	0	2 (0.3%)	2 (0.3%)
-Eastern Africa	0	16 (2.3%)	16 (2.0%)
Kenya	0	2 (0.3%)	2 (0.3%)
Rwanda	0	1 (0.1%)	1 (0.1%)
Somalia	0	2 (0.3%)	2 (0.3%)
South Sudan	0	1 (0.1%)	1 (0.1%)
Sudan	0	7 (1.0%)	7 (0.9%)
Tanzania	0	1 (0.1%)	1 (0.1%)
Uganda	0	1 (0.1%)	1 (0.1%)
-Southern Africa	0	7 (1.0%)	7 (0.9%)
Angola	0	5 (0.7%)	5 (0.6%)
Mozambique	0	1 (0.1%)	1 (0.1%)

Zambia	0	1 (0.1%)	1 (0.1%)

Country of birth	Freq (n)	Percent (%)	Cum.(%)
Angola	5	0.62	0.62
Benin	1	0.12	0.75
Burkina Faso	21	2.62	3.38
Burundi	1	0.12	3.50
Cape Verde	1	0.12	3.62
Cameroon	185	23.12	26.75
Chad	7	0.88	27.62
Congo	9	1.12	28.75
Ivory Coast	92	11.50	40.25
Ethiopia	1	0.12	40.38
Gabon	2	0.25	40.62
Gambia	18	2.25	42.88
Ghana	10	1.25	44.12
Guinea	141	17.62	61.75
Equatorial Guinea	42	5.25	67.00
Guinea-Bissau	15	1.88	68.88
Kenya	2	0.25	69.12
Liberia	1	0.12	69.25
Mali	94	11.75	81.00
Mauritania	4	0.50	81.50
Mozambique	1	0.12	81.62
Niger	4	0.50	82.12
Nigeria	23	2.88	85.00
Central African Republic	6	0.75	85.75
Democratic Republic of the Congo	1	0.12	85.88
Rwanda	1	0.12	86.00
Senegal	89	11.12	97.12
Sierra Leone	10	1.25	98.38
Somalia	2	0.25	98.62
Sudan	7	0.88	99.50

South Sudan	1	0.12	99.62
Tanzania	1	0.12	99.75
Uganda	1	0.12	99.88
Zambia	1	0.12	100.00
Total	800	100.00	

Suplementary Table S2. Characteristics of *Plasmodium* spp infection – TREATMENT

Treatment	Patients with malaria infection<br>N = 104
None	17 (16.3)
Malarone	52 (50.0)
Chloroquine	14 (13.5)
Mefloquine–Sulfadoxine	1 (1.0)
Artesunate	1 (1.0)
Artesunate–Sulfadoxine	1 (1.0)
Chloroquine–Primaquine	9 (8.7)
Dihydroartemisinin–Piperaquine	5 (4.8)
Malarone–Primaquine	4 (3.8)
